# Physical Approach to the Combustion Process of Polymer Fibre-Based Insulation Materials: An Initial Experimental Study

**DOI:** 10.3390/polym18151841

**Published:** 2026-07-27

**Authors:** Martin Sedlmajer, Jiri Zach, Jitka Peterkova, Vitezslav Novak, Azra Korjenic

**Affiliations:** 1 Faculty of Civil Engineering, Brno University of Technology, 612 00 Brno, Czech Republic; jiri.zach@vut.cz (J.Z.); jitka.peterkova@vut.cz (J.P.); vitezslav.novak1@vut.cz (V.N.); 2 Faculty of Civil and Environmental Engineering, TU Wien, Karlsplatz 13, 1040 Vienna, Austria; azra.korjenic@tuwien.ac.at

**Keywords:** combustion process, organic fibres, reaction to fire, flame propagation, fire safety

## Abstract

The flammability of building materials and products is expressed by the reaction to fire class according to the European standard EN 13501-1. However, the procedures described in this technical standard and in the related test standards do not fully reflect the physical and chemical principles of combustion. Especially in the area of lower classes B–F, the methodology is based on empirical principles, which makes it relatively very complicated to use the test results in the development of new insulation materials and predict their behaviour. This paper presents the initial phase of research that approaches combustion from a building-physics perspective at the scale of the fibre microstructure. In the case of insulation materials based on polymer fibres (very often based on recycled textile fibres), the situation is very complicated because, in most cases, the reaction to fire is given by combination of physical and chemical processes under contact of material with the flame. Rather than proposing a complete predictive model, this study formulates the basic governing relationships and reports the first experimental results, which will serve as the basis for a comprehensive model developed within the follow-up research. Single-flame-source tests (EN ISO 11925-2), combined with microstructural, calorimetric and airflow-resistivity measurements, established basic relationships between the observed flame behaviour and parameters such as fibre type, thickness and bulk density. The main observation is a clear ignition/no-ignition dichotomy between primary fibres (which melted and withdrew from the flame without igniting) and recycled fibres (which ignited), showing that neither the heat of combustion alone nor a purely physical description is sufficient—both physical and chemical aspects must be considered.

## 1. Introduction

The fire properties of building structures are crucial from a safety perspective, and their classification is governed by the European standards EN 13501-2 [1], which classifies structures in terms of fire resistance (the so-called REI classification), and EN 13501-1 [2], which classifies building materials in terms of their reaction to fire. Further divisions and requirements are contained in national technical standards; in the case of the Czech Republic, these are mainly ČSN 730802 and ČSN 73 0810 [3,4].

From the point of view of EN 13501-2, it is crucial to determine how long the structure can fulfil its function and prevent the spread of fire. As illustrated, for example, by the relatively poor fire performance of steel structures, high fire resistance is often not guaranteed even when materials classified as non-combustible according to EN 13501-1 are used (see below).

Within the classification according to EN 13501-1, building materials are divided into seven groups/classes (A1, A2, B, C, D, E, and F). For each reaction to fire class, a different testing mechanism is prescribed, which in practice considerably complicates development work, since the combustion process cannot be described in a single, unified way. The classification relies on a combination of tests according to EN ISO 11925-2, EN 13823, EN ISO 1182, and EN ISO 1716 [5,6,7,8] (the selection of tests and the test requirements and conditions depend on the class).

However, if the materials fall within the basic reaction-to-fire classes F and E, only the “single flame source test” according to EN ISO 11925-2 is required. This is the case for the vast majority of insulation materials based on polymer fibres. Based on the results of this test, preliminary conclusions are often drawn to determine whether a product can at least achieve class E, which is often a minimum requirement for use in building applications. The longer-term goal of this research is to develop a building-physics model capable of predicting the fire behaviour of fibrous insulation materials. As a first step, the present paper does not yet construct such a model; instead it establishes the basic governing relationships and reports initial experimental results, applying fire-development modelling principles at the microscopic scale of the material structure and drawing on established flame-height correlations (the Thomas formula and the Heskestad correlation [9,10]). The complete predictive model is the subject of the follow-up research.

Before a detailed description and understanding of the different behaviours of fibrous structures can be provided, it is essential to review the current state of knowledge on the insulation of polymeric fibrous materials and the methods used to assess their reaction to fire. Research in this area has so far followed two largely separate paths: the use of recycled textile fibres as a sustainable secondary raw material for thermal and acoustic insulation and the description of the thermal decomposition and combustion of the polymer fibres themselves.

Polyester is currently the most widely used textile fibre in the world—a position previously held by cotton—and in 2024 it represented 59% of global fibre production [11]. Polyesters (PES) constitute a group of synthetic polymers that contain an ester functional group within their macromolecules, with ester bonds comprising at least 85% of the linear chain. This group notably includes textile fibres based on polyethylene terephthalate (PET), polybutylene terephthalate (PBT), polyethylene naphthalate (PEN), and polytrimethylene terephthalate (PTT), which are primarily used in the textile industry. The basic raw material is crude oil, from which monomers are obtained—most commonly dimethyl terephthalate (or terephthalic acid) and a glycol. The stepwise polycondensation of these monomers—i.e., their gradual condensation alongside oligomers to form polycondensates—results in the formation of polyester itself. The most significant and widespread type is PET, from which a considerable volume of polyester fibre is produced, both from virgin and recycled materials. Throughout this paper, the term “PES” is used for the studied polyester fibres and insulation materials, while “PET” is used when referring to polymer-specific data for polyethylene terephthalate; since the studied PES fibres are PET-based, such data are considered representative of them. Polyester fibres are characterised by their lightfastness, resistance to weathering and microorganisms, and low moisture absorption, while their functional properties (such as shrinkage, pilling, or flammability) can be specifically adjusted through mechanical and chemical modifications [12].

The use of textile waste, particularly recycled polyester (PES) fibres, as a secondary raw material for building insulation has been extensively studied over the past decade. Patnaik et al. [13] demonstrated that mats combining waste wool and recycled PES fibres offer thermal and acoustic properties comparable to those of conventional mineral-based products, while Drochytka et al. [14] reached similar conclusions about insulation based primarily on waste PES fibres. Antolinc and Filipčič [15] produced thermal and acoustic insulation boards from industrial nonwoven PET textiles and treated selected samples with sodium silicate, specifically to improve their resistance to fire. These studies confirm the technical feasibility of this concept but also suggest that recycled fibres rarely behave the same as new material because their properties are influenced by the processing history, contamination, and a wide distribution of fibre lengths. At the same time, because PES fibres are flammable, their reaction to fire becomes an important design constraint. Flammable insulation can contribute significantly to the fire load of a building, and reviews on the fire-safe use of insulation materials repeatedly point out that the current classification framework, based on standardised “pass/fail” tests, provides only a limited understanding of the fundamental behaviour of the material and leaves little room for design optimisation [16].

From a fire reaction perspective, PES fibres present a particularly complex case. PET is a thermoplastic that shrinks, softens, and melts before ignition, and the reaction of the PES fibre to fire is therefore governed by a combination of physical phenomena—shrinkage, melting, formation of a melt zone, and potential dripping in flames—and the chemical decomposition of the polymer. Thermogravimetric studies of PET consistently show that it first melts at temperatures around 250 °C and that the main decomposition phase begins only above approximately 400 °C, with apparent activation energies reported on the order of 200 kJ/mol [17]. The reaction to fire is therefore the result of competing endothermic processes—heating, melting, and pyrolysis—and the exothermic combustion of volatile decomposition products. Petkova-Slipets et al. [18] demonstrated that the manufacturing method and surface treatment strongly influence flammability: adhesive-bonded nonwoven fabrics completely burnt, while silicone treatment protected the fibres and led to self-extinguishing behaviour with limited dripping. The enthalpy of fusion and the specific heat capacity, which together determine the endothermic portion of this energy balance, have been studied in detail for polyethylene terephthalate (PET): its equilibrium heat of fusion was determined calorimetrically [19], its specific heat capacity was measured in the relevant temperature range [20], systematic additive schemes allow the heat capacity of macromolecules to be estimated from the contributions of their structural groups [21], and the pyrolysis and combustion of organic fibres have generally been linked to their chemical composition [22].

A significant portion of research efforts have focused on improving the fire response of PES fibres through chemical means. Salmeia et al. [23] conducted a review of flame retardants based on phosphorus developed for fibres and textiles, which act in the condensed or gaseous phase and can be incorporated as comonomers during fibre formation or applied as surface treatments and coatings. Since PET is hydrophobic and chemically rather inert, surface treatments of PES fabrics often require an activation step; Ngo et al. [24] demonstrated that dielectric discharge plasma can increase the wettability of dyed PES fabric and thus improve the absorption and effectiveness of subsequent flame-retardant surface treatments. Such treatments are effective, but increase processing costs and complexity, can degrade the mechanical or aesthetic properties of the product, and raise environmental and health concerns—aspects that are particularly relevant for products made from recycled materials. Therefore, there is growing interest in achieving acceptable fire performance through the design of the fibre structure itself, with chemical flame retardants used only as a supplement, not as a primary measure.

Despite this body of work, it remains difficult to reliably predict fire behaviour based on material composition and structure. The Euroclass system of standard EN 13501-1 [2] classifies products essentially on an empirical basis, and for lower classes E and F, the single-flame test according to standard EN ISO 11925-2 [5] provides only a “pass/fail” result, which is difficult to relate to the microstructure of the material [25]. In contrast, heat transfer modelling for fibrous insulation has reached a considerable level of detail, although it has traditionally focused on the low-temperature regime, where conduction and radiation dominate and convection is usually neglected. Arambakam et al. [26], for example, simulated heat conduction and radiation in virtual three-dimensional fibrous structures and linked effective thermal conductivity to microstructural parameters such as fibre diameter, orientation, and packing density. However, these models focused primarily on optimising thermal insulation properties, not flammability of the material. In contrast, the combustion of fibrous organic insulation requires that heat and flame propagation be described on the scale of individual fibres, combining established correlations for flame height [9,10] with heat transfer through conduction, radiation, and convection. Experimental and theoretical studies of flame spread through combustible insulation confirm that flame height and spread rate are precisely determined by this interplay of heat transfer mechanisms [27], but such analyses have so far been conducted almost exclusively for closed-cell foams, not for fibrous materials. This approach, which directly links the microstructure of the material with its reaction to fire, has not yet been systematically applied to insulation made from recycled polymer fibres.

The contribution of the investigated problem lies primarily in the transfer of established flame-height correlations [9,10] and heat-transfer considerations to the scale of individual fibres and inter-fibre distances, i.e., to a scale orders of magnitude below their established macroscopic use, and conversely to temperatures far above the low-temperature regime of existing microstructural models of fibrous insulation [26]. A further contribution stems from the studied material class itself: air-permeable insulation made of recycled PES fibres, in which convection takes place inside the material and the polymer melt is free to move, differs fundamentally from the closed-cell foams for which flame spread through insulation has so far been analysed [27]. Beyond that, instead of a pass/fail classification, the combined microstructural, physical and combustion characterisation adopted here enables an exclusion-based analysis identifying which parameters do, and which do not, govern the ignition behaviour of the studied materials. This paper presents the initial phase of this approach; the development of a comprehensive predictive model is the subject of the follow-up research.

## 2. Materials

Various types of textile fibres were chosen for the research work, mainly because of their relatively high purity and also because of their practical importance with regard to the obligation to sort and subsequently recycle textiles within the EU from 2025.

These fibres were used for the production of insulation boards by the thermal bonding method. The boards were manufactured on a professional standard aerodynamic (air-lay) production line (Bematic, Bettarini & Serafini, Prato, Italy), using standard industrial processing: the base fibres were opened, blended and formed into a fibrous web aerodynamically, with the web layered to the area weights corresponding to the target bulk densities of 30, 50 and 70 kg/m^3^ at a board thickness of 60 mm. Polyester bicomponent (BiCo, Cetex-Rheinfaser, Ganderkesee, Germany) fibres with a fineness of 2.2 dTex were used as the thermal binder; their addition was 15% in all cases. The bonding temperature for the activation of the bicomponent fibres was 150 °C, i.e., above the melting point of the BiCo fibre sheath but well below the melting temperature of the PES core and of the base fibres, so that the bonding proceeds through the softened sheath only and the base fibres remain unaffected. Detailed line settings correspond to the standard production practice of the manufacturing line. The insulation materials were composed of four types of fibres. These were:

- primary PES fibres of various thicknesses—type A;
- secondary/recycled PES fibres—type B;
- modified secondary/recycled PES fibres with a flame-retardant treatment—type C;
- a mixture of secondary/recycled PES fibres and flakes generated during the recycling of filters and carpets—type D.

The fibres varied in fineness, ranging from 1.1 to 2.5 dTex. The fibres differed primarily in shape; for the secondary fibres, fibres with a high degree of defibration were selected, as well as a blend of fibres containing flocculent fibre clusters, which are typical of certain recycling technologies. The fibres were also verified to have been treated with a flame retardant. In total, six insulation materials were produced:

- Insulation No. 1—primary fine PES fibres (type A);
- Insulation No. 2—primary rough PES fibres (type A);
- Insulation No. 3—primary semi-rough PES fibres (type A);
- Insulation No. 4—high quality secondary fine PES fibres (type B);
- Insulation No. 5—high quality secondary fine PES fibres with flame retardant (type C);
- Insulation No. 6—secondary low quality PES fibres with flakes (type D).

**Figure 1:**
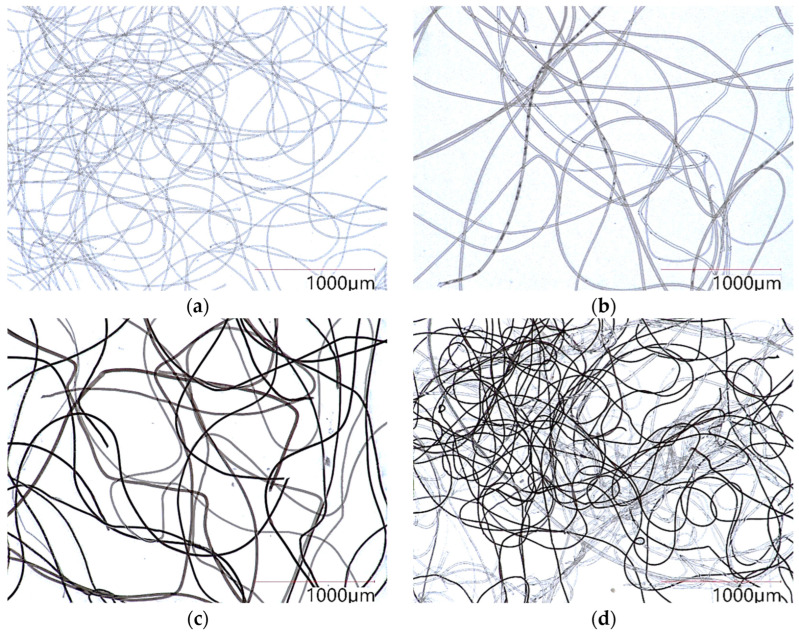

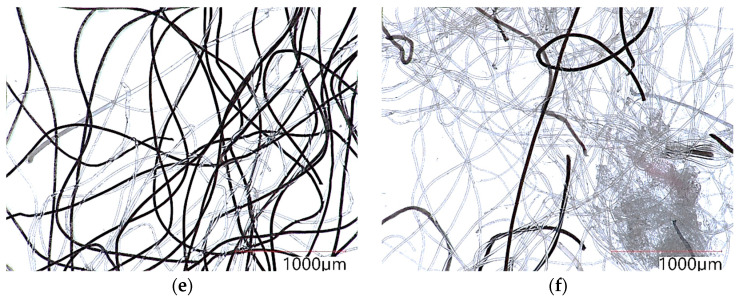
Images of insulations No. 1 through 6 from an optical microscope: (**a**) insulation No. 1—primary fine PES fibres; (**b**) insulation No. 2—primary rough PES fibres; (**c**) insulation No. 3—primary semi-rough PES fibres; (**d**) insulation No. 4—secondary fine high quality PES fibres; (**e**) insulation No. 5—high quality secondary fine PES fibres with flame retardant; and (**f**) insulation No. 6—low quality secondary PES fibres with flakes. The scale bar in each image is 1000 μm. The dark appearance of some fibres corresponds to their actual colour (black fibres in samples Nos. 3 and 5, grey fibres in samples Nos. 4 and 6), not to the imaging conditions.

For the experimental work, insulation materials were produced with nominal bulk densities of 30, 50 and 70 kg/m^3^. The photographs of individual insulators taken with an optical microscope are shown in Figure 1.

## 3. Methodology

A series of experiments were performed on the selected test samples (see Section 2 above) to determine their basic structural and physical properties, as well as other key properties from a combustion and reaction-to-fire perspective:

- Microstructural analysis: A macroscopic evaluation of the homogeneity and orientation of the fibres was initially performed. Subsequently, the thickness, length, and shape of the fibres were determined. Measurements were taken consistently from 20 fibres. If the insulation material comprised more than one type of fibre, measurements were taken for each type and the results were averaged, taking into account the weighted proportion of each type of fibre within the insulator. These measurements were conducted using a Keyence VHX-7000 (Keyence Corporation, Osaka, Japan) digital optical microscope, followed by scanning electron microscopy (SEM) using a TESCAN MIRA3 XMU electron microscope (TESCAN, Brno, Czech Republic). To describe fibre orientation, X-ray tomography was used, employing a Phoenix V|tome|x M300 tomograph (Waygate Technologies, Wunstorf, Germany).
- Physical properties: This involved measuring the thickness according to EN ISO 29466 [28], the bulk density according to EN ISO 29470 [29], and the heat of combustion according to EN ISO 1716. As an indirect, structural indicator of the potential contribution of convection within the insulation structure, the airflow resistivity was also measured according to EN ISO 9053-1 [30]. The airflow resistivity is a macroscopic measure of the viscous resistance of the fibre structure to air flow and is directly related to its air permeability; a higher resistivity indicates a structure in which the convective transport of air—and hence of oxygen and heat—is more restricted. Measurements were carried out on samples conditioned at an average temperature of +23 °C and a relative humidity of 50%. For each insulation variant (material × nominal bulk density), six specimens were cut from one produced board of identical parameters, and the bulk density was determined on all of them according to EN ISO 29470. From these, one representative specimen was selected for the subsequent detailed analyses; its actual measured bulk density is reported as the “real” value in in the results (Section 4). This design reflects the objective of this research phase: to relate the combustion behaviour to a specific, individually characterised fibre structure, in which the actual bulk density directly corresponds to the amount and arrangement of the fibres. The airflow resistivity was measured on three specimens taken from the same board. The heat of combustion of each fibre type was determined as the arithmetic mean of three measurements, using 0.1–0.2 g of fibres per measurement. Throughout the paper, the nominal bulk densities (30, 50 and 70 kg/m^3^) are used as level labels identifying the produced variants; the corresponding measured values of the representative specimens are given in Section 4.
- Combustion properties: The single-flame-source test was performed in accordance with EN ISO 11925-2 on the representative specimen of each variant (see above). The specimen dimensions were 250 × 90 mm (clause 5.2 of the standard) with a thickness of 60 mm, corresponding to the full thickness of the produced boards. The exposure conditions followed the standard: edge exposure according to clause 7.3.3.2.2, with a flame application time of 15 s and a total test duration of 20 s from the flame application. The primary parameters evaluated were whether ignition occurred (presence of sustained flaming, clause 3.6), whether the flame tip exceeded 150 mm above the flame application point, and whether the filter paper placed beneath the test specimen ignited. In addition, the following quantities were recorded:
  - time at which the flame tip exceeded 150 mm (clause 8.2 of the standard);
  - height of material degradation (melt-zone height; beyond the standard), defined as the maximum vertical extent of the visually identifiable zone of molten or degraded material, measured on the specimen after the test from the flame application point;
  - maximum flame height (clause 8.2 of the standard), i.e., the maximum vertical distance between the flame application point and the flame tip, reported below as the “flame spread height”;
  - time to reach the maximum flame spread height and the flame spread rate calculated therefrom (beyond the standard).

In deviation from clause 5.4 of EN ISO 11925-2, one specimen per variant was tested instead of the prescribed set of six, in line with the objective of linking the combustion behaviour to one individually characterised fibre structure; repeatability statistics are therefore not available at this stage and will be addressed within the follow-up research.

## 4. Results

As part of the basic characterisation of the insulators using optical and electron microscopy, the thickness and length of the fibres were determined for each insulator type. The resulting dimensions were then weighted according to the percentage of each type of constituent fibre within the insulators (e.g., raw and BiCo fibres).

**Figure 2:**
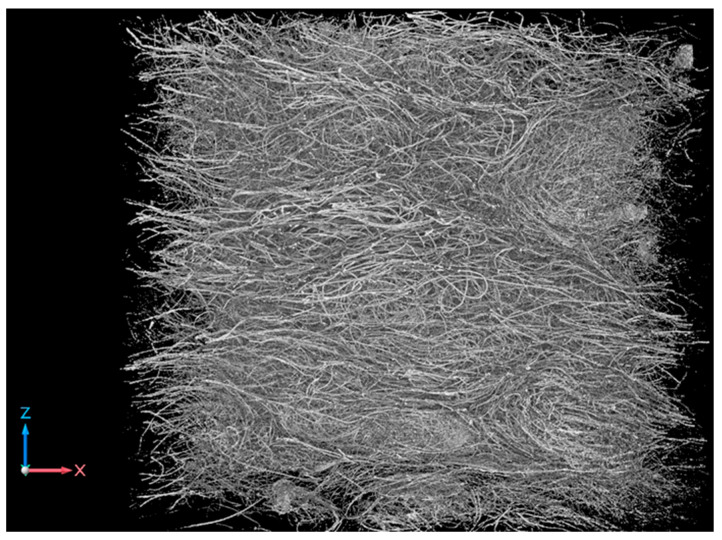
3D image from X-ray computed tomography (CT) of insulator No. 4 with irregular structure and low fibre orientation.

As the results indicate, insulators 1 and 4 exhibit the highest fineness, while insulator 2 contains the thickest fibres. However, it should be noted that all evaluated insulators generally feature very small fibre thicknesses, specifically within the range of approximately 14 to 25 µm. Regarding length, most insulators have an average fibre length between 25 and 55 mm. Structural homogeneity and fibre orientation were evaluated both macroscopically and by X-ray computed tomography (CT). The results of this structural evaluation are presented in Table 1 (a representative 3D structure of the insulators is illustrated in Figure 2).

As a simple numerical descriptor of morphological heterogeneity, the coefficient of variation (CV) of the fibre diameter is as follows:

CV = σ/d(1)

where *σ* is the standard deviation and *d* is the fibre diameter, which can be derived from Table 2: it ranges from approximately 3–7% for the primary and high-quality secondary fine fibres (insulations Nos. 1, 3, 4 and 5) to approximately 9% for insulation No. 2 and 12% for insulation No. 6, the latter confirming numerically the highest heterogeneity of the low-quality recycled fibres. With 20 measured fibres per insulation type, the 95% confidence half-width of the mean diameter remains within ±6% even for the most heterogeneous insulation, which is sufficient for the order-of-magnitude estimates derived from the mean dimensions in Section 5; the sample size is, however, not sufficient to characterise the complete dimensional distributions of the heterogeneous recycled fibres, which will be addressed by automated image analysis of the CT data within the follow-up research.

Furthermore, the physical properties important for the evaluation of insulation materials were determined. These included the dimensions, bulk density and heat of combustion, the latter being particularly important for the reaction to fire and fire behaviour in general. The results are summarised in Table 3.

As can be seen from the measured values, the heat of combustion did not differ substantially among the individual insulations. The highest value was measured for insulation No. 6, based on recycled PES fibres from carpets and filters, whereas the lowest values were measured for insulations Nos. 1 and 2, which contain pure PES fibres. The elevated value of insulation No. 6 (32.16 MJ/kg, compared with 21.3–21.4 MJ/kg for pure PES) indirectly indicates the presence of admixtures with a higher calorific value originating from the recycling feedstock; their chemical identification was, however, beyond the scope of this phase.

The ignitability of the insulation samples was further evaluated using the single-flame-source test. Beyond the standard requirements of EN ISO 11925-2, the overall behaviour of the test specimens was observed. In cases where ignition occurred, the flame spread rate was recorded and the depth of material degradation (“burn-through”) was assessed on the tested specimens after exposure. The results are summarised in Table 4.

In addition to the standard requirements, the time to reach 150 mm of flame spread and the time to reach the maximum flame spread height during the experiment were also monitored. These values were also used to calculate the flame spread rate; see Table 5.

From the results given in Table 4 and Table 5 it is clear that in samples Nos. 1–3 (based on primary fibres) the fibres did not ignite at all. However, the samples melted owing to the heat of the applied flame source. The melting height (melting zone) ranged from 56 mm to 107 mm. In all cases, these were samples based on primary PES fibres. The sample with coarser fibres exhibited a greater melt-zone height, which indicates a different spread of heat within the structure of the insulation (see below).

In the case of samples Nos. 4–6, which are based on recycled fibres, it can be seen that the insulators were ignited in all cases. The 150 mm limit was not reached. In the case of samples No. 4 and No. 6, the melting zone is lower than the flame spread height achieved. In the case of insulator No. 5, made from secondary PES fibres treated with a flame retardant, the insulator caught fire in all cases (similar to insulator No. 4), but the flame spread height was significantly lower and the area of melting of the insulator was significantly reduced.

The 150 mm limit was not exceeded by any sample, and the samples could therefore be classified as class E or better according to EN 13501-1.

In the case of the airflow resistivity s [kPa·s/m^2^] as defined in EN ISO 9053-1 [30], the specific airflow resistance was determined at low airflow velocities, and the results are presented in Table 6.

As can be seen from the measured values, the samples exhibit quite distinct behaviour. The measured trends in values as a function of bulk density indicate that airflow resistivity is influenced not only by fibre thickness, but also by fibre length and the overall internal structure of the insulation.

## 5. Evaluation of Results and Discussion

The central experimental finding of this study is the ignition/no-ignition dichotomy between the two fibre categories: none of the primary-fibre insulations (Nos. 1–3) ignited, while all recycled-fibre insulations (Nos. 4–6) did (Table 4 and Table 5); the following evaluation seeks the parameters and mechanisms behind this difference.

As part of the evaluation of the results obtained from the experiments (see Section 4 above), the average distance between the insulation fibres *d′* [μm] was estimated for the individual insulations from an idealised model of the fibre structure, in which the fibres are represented as parallel cylinders arranged in a square lattice. The fibre diameter *d* is the mean value determined by optical microscopy (Table 1). The linear density of a single fibre is:

ρ_f_ · (π · d^2^/4) (2)

where ρ_f_ = 1380 kg/m^3^ is the density of the PES fibre; the total fibre length per unit volume of the insulation follows from its bulk density *ρ_b_* as:

L_V_ = ρ_b_/(ρ_f_ · π · d^2^/4) (3)

The lattice spacing is then:

s = 1/√L_V_ (4)

and the clear distance between fibre surfaces is:

d′ = s − d(5)

The resulting values for the individual insulations and bulk densities are summarised in Table 7.

It is therefore clear from the results that these distances are very small and are easily bridged by the flame of a burning fibre. As can be seen, there is no direct correlation between the fire test results and the inter-fibre distances; however, if we compare only samples 4 and 6, which ignited, it is evident that the sample with thicker fibres exhibits slightly better values because the distance between the fibres is greater.

On the basis of the determined fibre properties, the theoretical flame height of a single burning fibre H_f_ [μm] was estimated using the Heskestad correlation [10] in the form:

H_f_ = 0.235 · Q^2/5^ − 1.02 · D (6)

where Q is the heat-release rate of the fibre in kW. The heat-release rate was obtained as:

Q = ṁ · ΔH_C_ (7)

with the mass burning rate

ṁ = (π · d^2^/4) · ρ_f_ · v_b_ (8)

where d is the measured mean fibre diameter (Table 1), ρ_f_ = 1380 kg/m^3^ is the density of the PES fibre, Δ*H_C_* is the measured gross heat of combustion (Table 3), and v_b_ = 1 mm/s is an assumed uniform linear burning velocity of the fibre (uniform fibre combustion). The term −1.02·*D* of the original correlation was neglected, since the source diameter *D* (equal to the fibre diameter, ~15–25 µm) is negligible compared with the resulting flame height. Because the Heskestad correlation was derived for turbulent, buoyancy-controlled diffusion flames of macroscopic sources, the calculated values should be regarded as order-of-magnitude estimates at the fibre scale; note, however, that *H_f_ ∝ v_b_^2/5^*, so the conclusion that the flame height greatly exceeds the inter-fibre distance is insensitive to the exact choice of *v_b_*.

The calculation results are shown in Table 8.

As can be seen from the calculated values, the flame heights (1790–2790 μm) are one to two orders of magnitude greater than the inter-fibre distances (43–122 μm, Table 7) for all six insulations. This means that once a single fibre ignites, its flame necessarily engulfs the neighbouring fibres, and the geometric condition for flame transfer within the fibre network is always satisfied with a large margin. The geometry of the fibre structure alone therefore cannot explain why some insulations ignited and others did not: if flame transfer between fibres were the limiting step, all samples would have ignited, whereas samples Nos. 1–3 did not ignite at all. The decisive factors must consequently be sought at the level of the individual fibre—in the chemical decomposition of the polymer and in the physical response of the material to the flame (melting, shrinkage and withdrawal of molten material; see below and the Conclusion). For the samples that ignited (Nos. 4–6), a weak positive relationship was observed between the calculated single-fibre flame height and the flame spread height measured according to EN ISO 11925-2, with a correlation coefficient of r = 0.57 (*n* = 9; *p* ≈ 0.11). Given the small number of materials, this should be regarded as an indicative trend only.

If we compare the values of the measured heats of combustion listed in Table 3 with the test results according to EN ISO 11925-2 listed in Table 4 and Table 5, we can conclude that the heat of combustion itself does not determine the test result. In the case of PES fibres, it is necessary to consider their melting at a temperature between 240 and 260 °C (depending on the specific type of fibre) and the associated consumption of latent heat of fusion, which amounts to up to 140 kJ/kg for fully crystalline PET [19]; real, partially crystalline fibres exhibit proportionally lower values, so this figure represents an upper bound. The thermophysical data of PET are used here as representative of the studied PES fibres, which are PET-based (see Introduction).

Therefore, not only the heat released during combustion but also the heat consumed must be considered. The heat consumed to bring 1 kg of fibres from the conditioning temperature to the ignition temperature was estimated as:

Q_cons_ = c_p_·ΔT + ΔH_m_ (9)

with the specific heat of PET c_p_ = 1.3 kJ/(kg·K) [20], treated as constant over the temperature range, and the ignition temperature of this fibre type of approximately 450 °C [31], consistent with the onset of the main decomposition phase of PET above approximately 400 °C [17]. This gives:

Q_cons_ = 1.3 · (450 − 23) + 140 = 555 + 140 (10)

where Q_cons_ ≈ 0.70 MJ/kg, an upper-bound estimate; note that the melting at 240–260 °C is an endothermic intermediate step within this heating path, not a competing value. The total heat obtainable from combustion of the same fibres is 21–32 MJ/kg under ideal combustion conditions—larger by a factor of roughly 30–45. From a purely energetic standpoint, the available energy is thus far more than sufficient for all the studied materials, for those that ignited and those that did not alike. This demonstrates that the energy balance cannot serve as a discriminating parameter: it is a necessary condition for ignition, not a sufficient one, since it contains neither the kinetics of heating nor the transport of material. Whether ignition actually occurs is decided at the fibre scale—above all by the behaviour of the melt and the ability of the fibre structure to retain the material in the heated zone (see below).

The combination of the results in Table 2 and Table 4 points to the decisive role of the melt behaviour at the fibre scale. The primary-fibre insulations (Nos. 1–3) consist of smooth, highly oriented fibres in a regular structure (Table 2, Figure 1a–c). Upon flame exposure, these fibres shrink and melt, and the molten material withdraws from the heated zone faster than it can be heated to ignition; this is documented by the combination of zero flame spread with extensive melt zones of 56–107 mm (Table 4). It should be stressed that the protective effect of melting is primarily geometric rather than energetic: the heat consumed by heating and fusion (≤0.70 MJ/kg, see above) is small compared with the heat of combustion, but the continuous withdrawal of the melt removes the fuel from the point of heating. In contrast, the recycled-fibre insulations (Nos. 4–6) form irregular, low-orientation structures containing entangled fibre clusters (Table 2, Figure 1d–f and Figure 2). The entanglement mechanically hinders the shrinkage and withdrawal of the melt; the material remains in the heated zone, reaches the ignition temperature and ignites. Consistently, for samples Nos. 4 and 6 the melt-zone height is smaller than the achieved flame spread height (Table 4). A similar governing role of the melt behaviour was reported by Petkova-Slipets et al. [18], where a surface treatment limiting dripping led to self-extinguishing behaviour of PES nonwovens. Minor impurities and contamination introduced during recycling may further contribute to the easier ignition of the secondary fibres; this contribution, however, remains a hypothesis, as no direct chemical characterisation was performed in this phase of the research.

**Figure 3:**
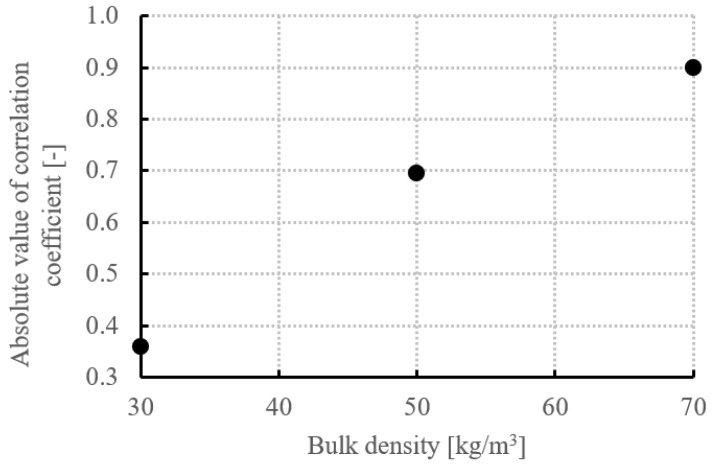
Absolute value |r| of the correlation coefficient between airflow resistivity and inter-fibre distance as a function of bulk density (the relationship is inverse: a larger inter-fibre distance corresponds to a lower resistivity).

Relatively interesting results were found regarding the relationship between the airflow resistivity values (Table 6) and the inter-fibre distance (Table 7). At each bulk density, the correlation coefficient was computed across all six insulation materials (*n* = 6). The relationship is inverse, as expected physically—a larger inter-fibre distance facilitates airflow through the structure and thus reduces the resistivity. The strength of the correlation increases with bulk density, from r = −0.36 (*p* ≈ 0.48) at 30 kg/m^3^ through r = −0.69 (*p* ≈ 0.13) at 50 kg/m^3^ to r = −0.90 (*p* ≈ 0.015) at 70 kg/m^3^; only the value at the highest bulk density is statistically significant at the 0.05 level, and the trend itself, being based on three density levels, should be regarded as indicative. The dependence of |r| on bulk density is shown in Figure 3.

Therefore, it is evident that at low bulk densities, the degree of structural order is a much more dominant factor (see Table 2). Conversely, at higher bulk densities, the structure becomes more highly ordered, and the airflow resistivity becomes strongly (inversely) correlated with the inter-fibre distance, which is itself governed by the fibre thickness. In the context of combustion, the airflow resistivity should be understood as an indirect, structural indicator of the potential contribution of convection: it quantifies how easily air—and with it oxygen and heat—can flow through the pore space of the insulation, but it is measured under isothermal, low-velocity conditions and does not capture buoyancy effects at fire temperatures. Notably, the present results indicate that the airflow resistivity does not govern ignition itself—the primary-fibre insulations did not ignite across the whole range of measured resistivities—and its role is expected to concern primarily the propagation stage. A direct quantification of the convective contribution therefore requires its experimental isolation, which is the aim of the planned reduced-pressure experiments.

Furthermore, the influence of bulk density on the combustion process was monitored for individual types of insulation. For samples Nos. 1–3 (based on primary PES), there was no ignition; therefore, only the melt-zone height can be compared. Here, it is clear, as mentioned above, that a higher value was achieved for sample No. 3.

In the case of samples Nos. 4–6 it is clear that the bulk density affects both the flame height and the melt zone height value.

**Figure 4:**
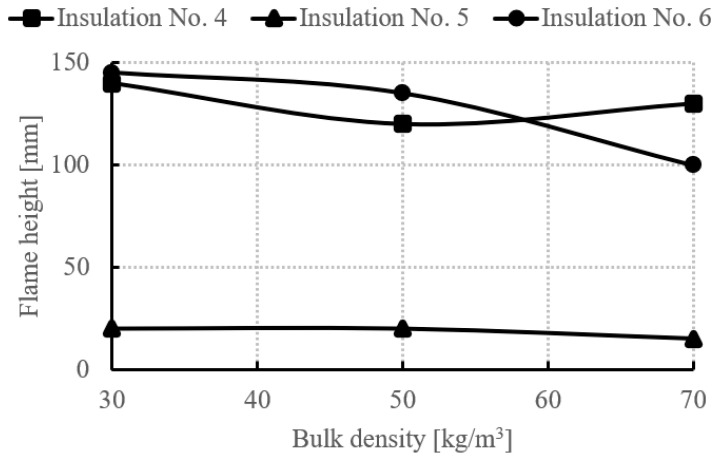
Dependence of the flame spread height on nominal bulk density for samples No. 4, 5, and 6.

Regarding the flame spread height, it can be observed that as the bulk density increases, the flame spread height decreases slightly in all cases, as illustrated in Figure 4. However, these differences are relatively minor and, therefore, the effect of the bulk density (within the observed range) is largely insignificant. It is evident that the presence of a free zone, oxygen availability, and heat transfer through convection and radiation are the dominant factors that influence the behaviour of a small flame; therefore, physical principles prevail. Although similar phenomena have been documented in other scientific studies, the varying focus and sample configurations in each case make it difficult to clearly isolate the individual effects described above [22,25,32].

## 6. Conclusions

The combustion behaviour of fibrous insulation materials proved to be highly complex, and predicting it solely from the composition and physical parameters of the insulation is exceedingly difficult. A very important finding of this initial study is the clear ignition/no-ignition dichotomy between the fibre types: the insulations based on primary fibres (Nos. 1–3) did not ignite at any bulk density—exhibiting zero flame spread combined with extensive melt zones of 56–107 mm—whereas all insulations based on secondary fibres (Nos. 4–6) ignited, with flame spread heights of 15–145 mm (Table 4). The experiments indicate that this difference is governed by the behaviour of the polymer melt in combination with the fibre arrangement: the smooth, highly oriented primary fibres shrink and melt away from the flame, and the withdrawal of the molten material prevents ignition, whereas the entangled clusters and low orientation of the recycled fibres (Figure 1f and Figure 2) mechanically hinder this withdrawal, so the material remains in the heated zone and ignites. Minor impurities introduced during recycling may contribute as a secondary factor; their role, however, remains to be verified by direct chemical characterisation. Not even pure PES recyclate thus exhibits a reaction to fire identical to that of virgin fibres, which highlights the challenge of processing municipal textile waste.

The supporting analyses showed that neither the heat of combustion nor the geometry of the fibre network can discriminate between the igniting and non-igniting materials: the theoretical flame height of a single fibre (thousands of micrometres, estimated using the Heskestad correlation as an order-of-magnitude value) exceeds the inter-fibre distances (tens of micrometres) by one to two orders of magnitude, so a burning fibre is always in direct contact with its neighbours. The governing phenomena therefore lie at the fibre scale—in the chemical decomposition of the polymer and the physical behaviour of the melt. Regarding the structure, insulators with thicker fibres (at identical bulk density) exhibited inferior behaviour owing to the larger inter-fibre distances, and the strength of the inverse correlation between airflow resistivity and inter-fibre distance increased with bulk density (only the value at 70 kg/m^3^, r = −0.90, being statistically significant), indicating improved structural ordering at higher densities; consistently, the poorest reaction to fire was exhibited by the samples with the lowest bulk density and thus the least ordered structure.

The ignitability results of this phase are based on single, individually characterised specimens per material and bulk density; quantification of repeatability on multiple replicates is part of the follow-up research.

From a practical point of view, it is important that the 150 mm limit of EN ISO 11925-2 was not exceeded by any of the tested samples, so even the recycled-fibre insulations could be classified as class E or better according to EN 13501-1. The results further indicate a realistic route towards improving the reaction to fire through the design of the fibre structure itself: adapting the bulk density to the fibre fineness, increasing the structural ordering and orientation of the fibres, and controlling the mobility of the polymer melt. Structural optimisation of this kind could reduce, or in favourable cases eliminate, the need for chemical flame retardants, which is particularly relevant for products based on recycled fibres.

The longer-term aim of the research is to develop a universal model enabling the optimisation of fibrous structures—ideally without, or with only minimal use of, chemical flame retardants. Within the follow-up research, it is planned to isolate the individual heat transfer processes—primarily convection, by testing under reduced pressure [33], complemented by thermal measurements—and to quantify the governing parameters into a comprehensive relationship describing the propagation of combustion at the microstructural level of organic-fibre insulation.

## Figures and Tables

**Table 1:** Description of the insulation structure.

Insulation	Colour	Description of the Structure	Description of Fibre Orientation
1	White	Regular	High orientation
2	White	Regular	High orientation
3	Black	Regular	High orientation
4	Grey	Irregular	Low orientation
5	Black	Irregular	Low orientation
6	Grey	Irregular	The lowest orientation

**Table 2:** Overview of fibre lengths and thicknesses for individual types of insulation.

Insulation	Thickness [Micron]		Length [mm]	
	Average	Deviation	Average	Deviation
1	14.74	1.02	34.32	3.52
2	24.28	2.22	49.45	7.07
3	20.23	0.70	54.55	8.14
4	14.64	1.14	24.04	2.30
5	15.30	0.81	38.87	3.34
6	20.96	2.50	32.11	3.90

**Table 3:** Overview of the physical properties of the insulators (real = measured bulk density of the representative specimen selected for detailed analysis).

Insulation	Combustion Heat	Bulk Density [kg/m^3^]	
	[MJ/kg]	Nominal	Real
1	21.42	30	35
		50	51
		70	71
2	21.32	30	32
		50	51
		70	69
3	25.17	30	33
		50	50
		70	72
	27.64	30	33
		50	50
		70	68
5	22.05	30	38
		50	49
		70	69
6	32.16	30	32
		50	52
		70	71

**Table 4:** Ignitability—test results for individual insulation samples in dependence on nominal bulk density.

Insulation	Nominal Bulk Density	Flame Spread Height	Height of the Melted Zone
	[kg/m^3^]	[mm]	[mm]
1	30	0	78
	50	0	68
	70	0	56
2	30	0	95
	50	0	95
	70	0	90
3	30	0	86
	50	0	107
	70	0	92
4	30	140	90
	50	120	80
	70	130	144
5	30	20	60
	50	20	58
	70	15	66
6	30	145	105
	50	135	95
	70	100	90

**Table 5:** Ignitability—additional results in dependence on nominal bulk density.

Insulation	Nominal Bulk Density	Time to Reach 150 mm Flame Spread	Time to Reach Maximum Flame Spread Height	Flame Spread Rate
	[kg/m^3^]	[s]	[s]	[m/s]
1	30	–	–	0.000
	50	–	–	0.000
	70	–	–	0.000
2	30	–	–	0.000
	50	–	–	0.000
	70	–	–	0.000
3	30	–	–	0.000
	50	–	–	0.000
	70	–	–	0.000
4	30	–	11	0.013
	50	–	15	0.009
	70	–	15	0.009
5	30	–	12	0.002
	50	–	14	0.001
	70	–	15	0.001
6	30	–	12	0.012
	50	–	14	0.010
	70	–	14	0.007

**Table 6:** Airflow resistivity [kPa·s/m^2^] of the test samples in dependence on nominal bulk density.

Sample/Nominal Bulk Density	30 kg/m^3^	50 kg/m^3^	70 kg/m^3^
1	2.4	10.0	26.6
2	4.4	8.4	14.8
3	5.0	9.8	15.5
4	7.7	15.3	26.3
5	8.3	17.9	29.3
6	3.9	6.7	13.4

**Table 7:** Overview of the fibre spacing *d′* [μm] for individual insulators and their nominal bulk density.

Sample/Bulk Density	30 kg/m^3^	50 kg/m^3^	70 kg/m^3^
1	73.9	53.9	43.3
2	121.8	88.8	71.3
3	101.4	74.0	59.4
4	73.4	53.5	43.0
5	76.6	55.9	44.9
6	105.0	76.6	61.5

**Table 8:** Overview of the calculated theoretical flame heights of a single fibre *Hf* [μm] for individual insulation materials.

Sample/Bulk Density	Theoretical Flame Height H_f_ [μm]
1	1790
2	2660
3	2460
4	1970
5	1860
6	2790

## Data Availability

The original contributions presented in this study are included in the article. Further inquiries can be directed to the corresponding author.

## References

[1] (2023). Fire Classification of Construction Products and Building Elements—Part 2: Classification Using Data from Fire Resistance Tests, Excluding Ventilation Services.

[2] (2018). Fire Classification of Construction Products and Building Elements—Part 1: Classification Using Data from Reaction to Fire Tests.

[3] (2023). Fire Protection of Buildings—Non-Industrial Buildings.

[4] (2016). Fire Protection of Buildings—General Requirements.

[5] (2020). Reaction to Fire Tests—Ignitability of Products Subjected to Direct Impingement of Flame—Part 2: Single-flame Source Test.

[6] (2022). Reaction to Fire Tests for Building Products—Building Products Excluding Floorings Exposed to the Thermal Attack by a Single Burning Item.

[7] (2020). Reaction to Fire Tests for Building Products—Non-Combustibility Test.

[8] (2018). Reaction to Fire Tests for Products—Determination of the Gross Heat of Combustion (Calorific Value).

[9] Thomas P.H. (1963). The size of flames from natural fires. Symposium (International) on Combustion.

[10] Heskestad G. (1983). Luminous heights of turbulent diffusion flames. Fire Saf. J..

[11] Textile Exchange (2025). Materials Market Report 2025.

[12] Sattler H., Schweizer M. (2011). Fibres, 5. Polyester Fibres. Ullmann’s Encyclopedia of Industrial Chemistry.

[13] Patnaik A., Mvubu M., Muniyasamy S., Botha A., Anandjiwala R.D. (2015). Thermal and sound insulation materials from waste wool and recycled polyester fibres and their biodegradation studies. Energy Build..

[14] Drochytka R., Dvořáková M., Hodná J. (2017). Performance evaluation and research of alternative thermal insulation based on waste polyester fibres. Procedia Eng..

[15] Antolinc D., Filipčič K.E. (2021). Recycling of Nonwoven Polyethylene Terephthalate Textile into Thermal and Acoustic Insulation for More Sustainable Buildings. Polymers.

[16] Hidalgo J.P., Welch S., Torero J.L. (2015). Performance criteria for the fire safe use of thermal insulation in buildings. Constr. Build. Mater..

[17] Alhulaybi Z., Dubdub I. (2023). Comprehensive Kinetic Study of PET Pyrolysis Using TGA. Polymers.

[18] Petkova-Slipets R., Zlateva P., Staneva D. (2022). Influence of the polyester non-wovens production type on their thermal and flammability properties. J. Eng. Appl. Sci..

[19] Mehta A., Gaur U., Wunderlich B. (1978). Equilibrium melting parameters of poly(ethylene terephthalate). J. Polym. Sci. Polym. Phys. Ed..

[20] Smith C.W., Dole M. (1956). Specific heat of synthetic high polymers. VII. Polyethylene terephthalate. J. Polym. Sci..

[21] Gaur U., Cao M.-Y., Pan R., Wunderlich B. (1986). An addition scheme of heat capacities of linear macromolecules. Carbon backbone polymers. J. Therm. Anal..

[22] Dorez G., Ferry L., Sonnier R., Taguet A., Lopez-Cuesta J.M. (2014). Effect of cellulose, hemicellulose and lignin contents on pyrolysis and combustion of natural fibres. J. Anal. Appl. Pyrolysis.

[23] Salmeia K.A., Gaan S., Malucelli G. (2016). Recent Advances for Flame Retardancy of Textiles Based on Phosphorus Chemistry. Polymers.

[24] Ngo H.-T., Vu Thi H.K., Nguyen T.-B. (2021). Surface Modification by the DBD Plasma to Improve the Flame-Retardant Treatment for Dyed Polyester Fabric. Polymers.

[25] Fangrat J. (2017). On non-combustibility of commercial building materials. Fire Mater..

[26] Arambakam R., Vahedi T.H., Pourdeyhimi B. (2013). A simple simulation method for designing fibrous insulation materials. Mater. Des..

[27] Ma X., Tu R., Cheng X., Zhu S., Ma J., Fang T. (2018). Experimental Study of Thermal Behavior of Insulation Material Rigid Polyurethane in Parallel, Symmetric, and Adjacent Building Façade Constructions. Polymers.

[28] (2022). Thermal Insulating Products for Building Applications—Determination of Thickness.

[29] (2020). Thermal Insulating Products for Building Applications—Determination of the Apparent Density.

[30] (2018). Acoustics—Determination of Airflow Resistance—Part 1: Static Airflow Method.

[31] Levchik S.V., Weil E.D. (2004). A review on thermal decomposition and combustion of thermoplastic polyesters. Polym. Adv. Technol..

[32] Zachova P., Zach J., Toserova I., Novak V. (2020). Development of advanced thermal and acoustic insulation materials based on recycled PES fibres. International Multidisciplinary Scientific GeoConference Surveying Geology and Mining Ecology Management, SGEM.

[33] Zach J., Novak V., Peterkova J., Bubenik J. (2020). Development of Vacuum Insulation Panels with Utilization of Organic By-Products. Energies.

